# Influence of a humidification system on ventilator-associated pneumonia: a randomized controlled trial

**DOI:** 10.1186/cc12656

**Published:** 2013-06-19

**Authors:** JRA de Azevedo, A da Luz Leitão, NNP Souza, MP Pereira

**Affiliations:** 1 Hospital São Domingos, Bequimão, São Luis, MA, Brazil

## Introduction

Heat and moisture exchanger (HME) filters are replacing heated humidifiers (HH) to promote humidification of inhaled air during mechanical ventilation. The argument is often that the HMEs are related to a lower incidence of VAP. On the other hand, a restriction to HMEs is that they are associated with an increased incidence of obstruction of the endotracheal tube. It is now known that the ventilator circuit and its accessories, including the humidification system, have very little impact on the incidence of VAP. The aspiration of contaminated secretions is the main determinant of this complication. The objective of this study was to compare the incidence of VAP in patients undergoing mechanical ventilation using HH versus HME filter. Secondarily, we analyzed the incidence of tracheal tube obstruction in both groups.

## Methods

This prospective randomized study included all adult patients undergoing mechanical ventilation for more than 12 hours, admitted to a 37-bed general ICU in the period from October 2011 to January 2013. The study was approved by the Ethics Committee of Hospital São Domingos under number 026/ 2011. Group 1 used HME filters TWINSTAR 55 (Drager Medical, Germany). The filter was changed every 24 hours. Group 2 used heated humidifier MR 730 (Fisher and Paykel Healthcare Inc., Auckland, New Zealand). The humidifier was exchanged between patients with the ventilator circuit.

## Results

One hundred and fifty-three patients were randomized. Two were excluded: excessive airway secretion forcing frequent exchanges of HME filter (G1); length of MV <12 hours (G2). One hundred and fifty-one patients were analyzed. The two groups were comparable with regard to demographic data and severity (APACHE IV and SOFA). The main indication of MV was hypoxemic respiratory failure (G1 = 34; G2 = 35). The duration of mechanical ventilation was comparable between the two groups (G1 = 10.6 ± 19.0 days; G2 = 12.0 ± 20.7 days; *P *= 0.65). The incidence of VAP also did not differ significantly between groups: G1 = 7 (9.2%), G2 = 10 (13.3%); *P *= 0.42. No endotracheal tube occlusion was identified.

## Conclusion

This study, like several others, including a meta-analysis of the Cochrane Institute (2,236 patients), failed to show that HME filters are able to reduce the incidence of ventilator associated pneumonia, compared with heated humidifiers. We had no cases of obstruction of the endotracheal tube. There is evidence in the literature that an HME filter change at intervals greater than that we used are related to a higher incidence of artificial airway occlusion. The choice of the system of humidification of inspired air in patients on mechanical ventilation (heated humidifier filter or HME) should not have the purpose to reduce the incidence of VAP.

